# Utility of ultra-sensitive qPCR to detect *Plasmodium falciparum* and *Plasmodium vivax* infections under different transmission intensities

**DOI:** 10.1186/s12936-020-03374-7

**Published:** 2020-09-03

**Authors:** Maria Gruenberg, Clara Antunes Moniz, Natalie E. Hofmann, Cristian Koepfli, Leanne J. Robinson, Elma Nate, Wuelton Marcelo Monteiro, Gisely Cardoso de Melo, Andrea Kuehn, Andre M. Siqueira, Wang Nguitragool, Quique Bassat, Marcus Lacerda, Jetsumon Sattabongkot, Ivo Mueller, Ingrid Felger

**Affiliations:** 1 Swiss Tropical and Public Health Institute, Basel, Switzerland; 2 University of Basel, Basel, Switzerland; 3 Walter and Eliza Hall Institute of Medical Research, Melbourne, Australia; 4 Papua New Guinea Institute of Medical Research, Madang, Papua New Guinea; 5 Fundação de Medicina Tropical Dr. Heitor Vieira Dourado (FMT-HVD), Manaus, Brazil; 6 Instituto Nacional de Infectologia Evandro Chagas, Fiocruz, Rio de Janeiro, Brazil; 7 Department of Molecular Tropical Medicine & Genetics, Faculty of Tropical Medicine, Mahidol University, Bangkok, Thailand; 8 ISGlobal, Hospital Clínic - Universitat de Barcelona, Barcelona, Spain; 9 Universidade Do Estado Do Amazonas, Manaus, Brazil; 10 Mahidol Vivax Research Unit, Faculty of Tropical Medicine, Mahidol University, Bangkok, Thailand; 11 Department of Medical Biology, University of Melbourne, Melbourne, Australia; 12 Present Address: Eck Institute for Global Health, University of Notre Dame, Notre Dame, IN USA; 13 Present Address: Burnet Institute, Melbourne, Australia; 14 Present Address: Malaria Parasite & Hosts Unit, Institut Pasteur, Paris, France

**Keywords:** Ultra-sensitive, qPCR, Molecular diagnostics, Low-density, varATS, mtCOX1

## Abstract

**Background:**

The use of molecular diagnostics has revealed an unexpectedly large number of asymptomatic low-density malaria infections in many malaria endemic areas. This study compared the gains in parasite prevalence obtained by the use of ultra-sensitive (us)-qPCR as compared to standard qPCR in cross-sectional surveys conducted in Thailand, Brazil and Papua New Guinea (PNG). The compared assays differed in the copy number of qPCR targets in the parasite genome.

**Methods:**

*Plasmodium falciparum* (*Pf*) and *Plasmodium vivax* (*Pv*) parasites were quantified by qPCR amplifying the low-copy *Pf_* and *Pv*_18S rRNA genes or the multi-copy targets *Pf*_varATS and *Pv*_mtCOX1. Cross-sectional surveys at the three study sites included 2252 participants of all ages and represented different transmission intensities.

**Results:**

In the two low-transmission areas, *P. falciparum* positivity was 1.3% (10/773) (Thailand) and 0.8% (5/651) (Brazil) using standard *Pf*_18S rRNA qPCR. In these two countries, *P. falciparum* positivity by *Pf_*varATS us-qPCR increased to 1.9% (15/773) and 1.7% (11/651). In PNG, an area with moderate transmission intensity, *P. falciparum* positivity significantly increased from 8.6% (71/828) by standard qPCR to 12.2% (101/828) by us-qPCR. The proportions of *P. falciparum* infections not detected by standard qPCR were 33%, 55% and 30% in Thailand, Brazil and PNG. *Plasmodium vivax* was the predominating species in Thailand and Brazil, with 3.9% (30/773) and 4.9% (32/651) positivity by *Pv*_18S rRNA qPCR. In PNG, *P. vivax* positivity was similar to *P. falciparum*, at 8.0% (66/828). Use of *Pv*_mtCOX1 us-qPCR led to a significant increase in positivity to 5.1% (39/773), 6.4% (42/651) and 11.5% (95/828) in Thailand, Brazil, and PNG. The proportions of *P. vivax* infections missed by standard qPCR were similar at all three sites, with 23%, 24% and 31% in Thailand, Brazil and PNG.

**Conclusion:**

The proportional gains in the detection of *P. falciparum* and *P. vivax* infections by ultra-sensitive diagnostic assays were substantial at all three study sites. Thus, us-qPCR yields more precise prevalence estimates for both *P. falciparum* and *P. vivax* at all studied levels of endemicity and represents a significant diagnostic improvement. Improving sensitivity in *P. vivax* surveillance by us-qPCR is of particular benefit, because the additionally detected *P. vivax* infections signal the potential presence of hypnozoites and subsequent risk of relapse and further transmission.

## Background

Asymptomatic *Plasmodium* infections, persisting undetected in the population, present a silent reservoir for on-going transmission and pose a major challenge in malaria elimination [1]. Parasite densities in asymptomatic infections are often low, and therefore infections remain undetected by light microscopy (LM) or rapid diagnostic tests (RDT), the standard diagnostics to control malaria in endemic countries. In epidemiological studies, quantitative PCR (qPCR) has increasingly been used to obtain more precise prevalence rates for *Plasmodium spp.* infections [2–6]. The most widely used qPCR assays for detection of *Plasmodium* species target genus- or species-specific regions within the 18S rRNA genes [2, 7–9]. The genomes of *Plasmodium falciparum* (*Pf*) and *P. vivax* (*Pv*) harbour 5 and 3 copies of 18S rRNA genes, however, some copies show substantial sequence variation so that primers and probes for *P. vivax* target only one copy of this gene family [10]. Comparison of the widely used 18S rRNA assays with ultra-sensitive detection methods uncovered a 2–4 times higher parasite prevalence of low-density malaria infections at medium and low transmission settings that previously remained undiagnosed by less sensitive detection tools [6, 11, 12].

Enhanced detection sensitivity by qPCR can be achieved in three ways: firstly, by processing larger volumes of blood and concentrating the DNA during the extraction procedure [11, 13], secondly, by using ultra-sensitive-qPCR (us-qPCR) assays that target multi-copy sequences in the parasite genome [10, 11, 14–16] instead of a single-copy or low-copy gene, and thirdly, by detecting highly abundant 18S rRNA transcripts by quantitative reverse-transcriptase PCR (qRT-PCR) instead of rDNA. Collecting a large volume of blood by venipuncture instead of finger prick is mostly not feasible for large surveys. RNA should be preserved using specific buffers and stored at − 80 °C to avoid degradation. Compared to DNA-based tests, RNA extraction and qRT-PCR are more costly and the handling and amplification of the high copy 18S rRNA transcripts are more prone to contamination [2, 10]. Both these methods are, therefore, less practicable for malaria surveillance. In contrast, amplifying multi-copy genes by qPCR improves diagnostic sensitivity even when using finger prick blood samples [11]. For ultra-sensitive detection of low-density *P. falciparum* infections, the *Pf*_varATS us-qPCR was developed that targets the acidic terminal sequences of multi-copy *var* genes [11]. For low-density *P. vivax* infections, *Pv*_mtCOX1 us-qPCR targeting the *cytochrome oxidase 1* gene was designed [10]. COX1 is encoded in the mitochondrial genome and is present in at least 20 copies per cell [17]. Both us-qPCR assays for *P. falciparum* and *P. vivax* detect 10-fold lower parasite densities as compared to *Pf*_ and *Pv*_18S rRNA assays [10, 11].

Ultra-sensitive diagnostic methods are of particular importance for *P. vivax*, as this species generally presents lower levels of parasitemia compared to *P. falciparum* due to its preferential invasion of the reticulocyte, rather than the mature red blood cell. For example in Papua New Guinea (PNG), where both species are equally prevalent, mean *P. vivax* parasite densities by LM in community samples were seven times lower than those of *P. falciparum* [3]. In the context of elimination, malaria diagnostics may require detection of the full extent of carriers of both parasite species. This is of particular relevance for *P. vivax*, as individuals with an undetected *P. vivax* infection may carry dormant hypnozoites and thus present a future reservoir for onward transmission due to later relapses.

Over the last decade, malaria epidemiological studies have increasingly employed molecular diagnostics, and PCR-based prevalence rates became available from many endemic areas [1, 16]. Meta-analyses have investigated the relationship of PCR and LM prevalence for *P. falciparum* and *P. vivax* [1, 16, 18]. For *P. falciparum* this analysis has shown that the proportion of submicroscopic *P. falciparum* infections greatly varied according to *P. falciparum* transmission intensity and ranged from 20% in areas of high transmission to 80% in low endemicity settings [1]. For *P. vivax*, two systematic reviews analysed cross-sectional studies from Asia, South America and South Pacific and gave concordant results [16, 18], showing that PCR performed in community samples detected on average more than twice as many infections as LM. Similar to *P. falciparum,* the proportion of submicroscopic *P. vivax* infections was negatively correlated with prevalence by LM, i.e. a larger proportion of submicroscopic *P. vivax* infections was observed in low compared to high *P. vivax* transmission areas [16].

Together these earlier studies indicated that in areas of low malaria endemicity, molecular diagnostic methods are of particular importance for assessing the transmission reservoir and for informing malaria control interventions [1, 16]. The hypothesis for the current study was that us-qPCRs, compared to standard qPCRs, could also be particularly beneficial in low endemic areas, as they could further improve the precision of molecular prevalence estimates. The gain in additionally diagnosed parasite carriers by us-qPCR might also vary according to transmission intensity, similar to the described larger proportion of submicroscopic *P. falciparum* infections found in low endemic settings [1, 16]. This study explored the advantage of using us-qPCR for *P. falciparum* and *P. vivax* compared to diagnosis by standard 18S rRNA qPCR. By analysing community samples from low transmission settings in Brazil and Thailand and a setting of moderate transmission in PNG, the variation of gains across the study sites was examined, as well as the age distribution of submicroscopic infections by standard *versus* us-qPCR.

## Methods

### Study sites and archived samples

This study used 2252 archived DNA samples from cross-sectional blood collections in Thailand, Brazil and PNG between 2012 and 2014. A detailed description of these three study sites and results by standard qPCR were published previously [4, 5, 19] (W. Monteiro, pers. commun.). Sampling procedures, DNA extraction from red blood cell pellets and diagnostic tests were harmonized among all sites. DNA samples from participants of all age-groups were selected randomly from larger subsets from Thailand and Brazil. 773 archived DNA samples from Thailand originated from a low-endemic village of the BongTi region, Kanchanaburi province, and were collected in 2012. The 651 archived DNA samples from Brazil were collected in the Manaus region in March 2014. From PNG, the full sample sets (828 archived samples) from the two communities Megiar and Utu, one of high and one of low transmission, were analysed. These cross-sectional surveys were conducted between May and July 2014 in Madang province. Prior to performance of us-qPCR, good quality of the stored DNA was confirmed by repeating species-specific *Pf_* and *Pv*_18S rRNA qPCR assays in a minimum of 150 samples from each study site. Results in these repeated subsets were compared to the first round of *Pf*_ and *Pv*_18S rRNA assays [4, 5, 19]. For Thailand and Brazil complete overlap for *P. falciparum*-positivity and almost complete overlap for *P. vivax*-positivity was found. For PNG *P. falciparum*-positivity agreed very well, but discrepancies were observed for *P. vivax* between first and repeat results. Therefore, all PNG samples were re-analysed by *Pv*_18S rRNA qPCR and only the data from repeats were used for the comparison of diagnostic methods.

### qPCR and us-qPCR

The three sets of samples were analysed in their respective countries of origin. For quantification of *P. falciparum* parasites in sample sets from Thailand and Brazil, a dilution of the WHO International Standard “*Plasmodium falciparum* DNA for NAT Assays” (NIBSC, UK) was used. For quantification of *P. falciparum* parasites in the PNG sample set, a serial dilution of cultured 3D7 parasites was used. *Plasmodium vivax* samples were quantified using a standard curve generated from dilutions of *P. vivax* control plasmids containing inserts corresponding to the respective qPCR or us-qPCR target sequences [2, 10].

*Pf*_ and *Pv*_18S rRNA qPCR was performed at all sites with the same protocols [2]. The same applies for *Pf*_varATS and *Pv*_mtCOX1 us-qPCR assays [10, 11], the performance of which is summarized in Table 1. For a comparison of parasite densities between study sites, qPCR efficiency and sensitivity of each assay was analysed across laboratories (Additional file 1: Table S1, Additional file 2: Fig. S1)**.** Except for 5 Brazilian samples that were initially positive by *Pf*_18S rRNA qPCR, sufficient DNA for us-qPCR was available for all samples. In the comparative analysis of overall prevalence by varATS qPCR across the three sites, these 5 Brazilian samples were included and considered positive, justified by their original *Pf*_18S rRNA qPCR positivity and by data from Thailand and PNG, where 100% (Thailand) or 98% of 18S rRNA-positives (PNG) were also positive by varATS qPCR (Additional file 2: Fig. S2). In the analysis of parasite densities, the quantification of these 5 samples were based on the earlier *Pf*_18S rRNA qPCR data.

**Table 1:** Molecular diagnostic assays used in this study

Molecular assays	Target gene	Amplicon length (bp)	Target copies/parasite genome	LOD^a^ (parasite/µL)	References
*P. falciparum* qPCR					
*Pf*_18S rRNA	18S rRNA genes	221	3	1.57	[2]
*Pf_*varATS	*var* genes	65	≥ 59	0.06–0.15	[11]
*P. vivax* qPCR					
*Pv_*18S rRNA	18S rRNA gene	221	1	0.13–0.92	[2, 12]
*Pv*_mtCOX1	*mtCOX1*	148	> 20	0.01–0.023	[10, 12]

^a^LOD for *Pf*_varATS qPCR determined by a serial dilution of the WHO International Standard (presented as parasites/µL whole blood, reconstituted using WHO international standard together with uninfected blood); LOD for *Pv*_mtCOX1 determined in two trendlines generated from LM-quantified field samples (presented as parasites/µL whole blood from field samples)

### Data analysis

Data were analysed using R.Studio version 3.5.1. Parasite densities determined by standard qPCR and us-qPCR assays were log10 transformed and presented as median densities, if not stated otherwise. A febrile malaria case was defined as an individual with fever or history of fever (body temperature ≥ 37.5 °C at blood sampling or within the preceding 2 days) and microscopic detection of parasites. Proportions of samples positive by standard 18S rRNA qPCR and us-qPCR were compared using McNemar´s Chi^2^ test. A linear regression model was used to evaluate the difference of proportional gains by us-qPCR among the 3 study sites.

## Results

### *Plasmodium falciparum*-positivity by qPCR and us-qPCR

**Fig. 1:**
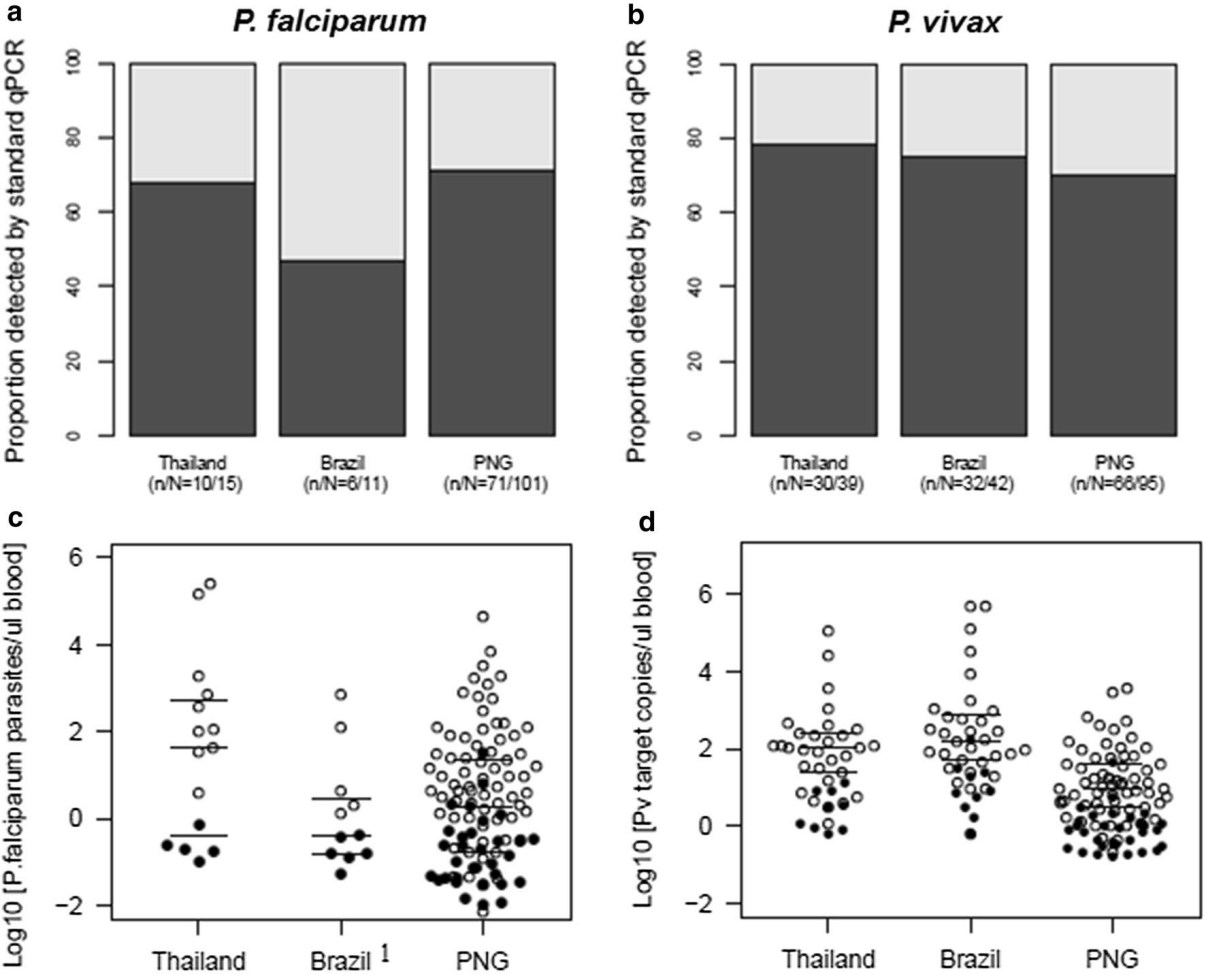
Proportion of *P. falciparum* and *P. vivax* infections detected by qPCR *versus* us-qPCR by country. Proportion of *P. falciparum* (**a**) and *P. vivax* infections (**b**) positive by species-specific 18S rRNA qPCR among all samples positive by any qPCR assay (standard qPCR or us-qPCR) and parasite densities of *P. falciparum* (**c**) and *P. vivax* infections (**d**) detected by 18S rRNA qPCR versus only by us-qPCR. White circles: infection detected by standard qPCR; black circles: infection detected only by us-qPCR. ^1^Owing to exhaustion of DNA, the parasite densities of 5 Brazilian samples (white circles) were quantified by 18S rRNA qPCR

By standard *Pf*_18S rRNA qPCR, positivity was significantly higher in PNG compared to the other field sites (Chi^2^–test, p < 0.001). The absolute number of additionally detected *P. falciparum* infections by us-qPCR was highest in PNG, the site with intermediate transmission intensity. The relative proportion of additionally detected *P. falciparum* infections differed substantially between Brazil (55%, 95% CI 25–82) and Thailand (33%, 95% CI 13–61) and PNG (30%, 95% CI 21–40) (Table 2, Fig. 1a, c), but owing to the small number of *P. falciparum* samples at the sites of low endemicity, these differences in gains were not statistically significant (p = 0.77).

**Table 2:** *P. falciparum* and *P. vivax* positivity in community samples from Thailand, Brazil and PNG, determined by species-specific 18S rRNA (qPCR) *versus* ultra-sensitive qPCR (us-qPCR)

	*P. falciparum* positivity (%)			*P. vivax* positivity (%)		
	qPCR (95% CI) (n/N)	us-qPCR (95% CI) (n/N)	*p*-value^a^	qPCR (95% CI) (n/N)	us-qPCR (95% CI) (n/N)	*p*-value^a^
Thailand	1.3% (0.7–2.5) (10/773)	1.9% (1.1–3.3) (15/773)	0.074	3.90% (2.7–5.6) (30/773)	5.00% (3.7–6.9) (39/773)	0.008
Brazil	0.80% (0.3–1.9) (5/651)	1.70% (0.9–3.1) (11/651) (11/651)	0.041	4.90% (3.4–6.9) (32/651)	6.50% (4.7–8.7) (42/651)	0.004
PNG	8.60% (6.8–10.7) (71/828)	12.20% (10.1–14.7) (101/828)	< 0.001	8.00% (6.3–10.1) (66/828)	11.50% (9.4–13.8) (95/828)	< 0.001

^a^McNemar’s Chi^2^-test used to determine if gain of infections by us-qPCR was significant

### *Plasmodium vivax* prevalence by qPCR and us-qPCR

*Plasmodium vivax* was the prevailing species in Thailand and Brazil with 3.9% (30/773) and 4.9% (32/651) *Pv*_18S rRNA-positive individuals. *Pv*_18S rRNA positivity at these field sites was significantly lower than in PNG (8.0%, 66/828, Chi^2^-test, p < 0.001) (Table 2), where *P. vivax* and *P. falciparum* prevalence rates were comparable (Table 2). The absolute number of *P. vivax* infections that were additionally detected by *Pv*_mtCOX1 us-qPCR was highest in PNG (Table 2), which in global comparison represents particularly high *P. vivax* prevalence. The proportion of *P. vivax* infection additionally detected by us-qPCR was similar in low endemic Thailand (23%, 95% CI 12–40) and Brazil (24%, 95% CI 13–40), but was higher in PNG (31%, 95% CI 22–41) (Fig. 1b). By us-qPCR *P. falciparum*/*P. vivax* co-infections were detected in 4.1% (34/828) individuals from PNG and in 0.6% (5/773) individuals from Thailand, whereas no co-infection was observed in Brazil.

### *Plasmodium falciparum* and *P. vivax* densities

Quantification by us-qPCR was reliable as shown by a good correlation with quantification by 18S rRNA qPCR (Additional file 2: Fig. S3). Characteristics of both us-qPCR assays that are relevant for quantification, agreed well between the different laboratories (Additional file 1: Table S1, Additional file 2: Fig. S1), hence, between-site comparisons of parasite densities by us-qPCR are appropriate. In contrast, densities of *P. falciparum* and *P. vivax* by us-qPCR cannot be compared directly for several reasons: Firstly, both multi-copy assays target different numbers of gene copies per genome, thus, a comparison based on numbers of detected gene copies/µL would be disproportionate. Secondly, *P. falciparum* densities were reported as parasites/µL blood by converting Ct-values determined by *Pf*_varATS us-qPCR into parasite numbers using a parasite trendline, whereas for *P. vivax*, owing to the lack of in vitro culture, a well-defined *P. vivax* parasite trendline was not available for absolute parasite quantification. Instead, *P. vivax* densities were derived from a dilution series of a plasmid standard and reported as *Pv*_mtCOX1 copy numbers/µL blood. While densities of *P. falciparum* and *P. vivax* are not comparable between species, the median densities of either species are well comparable between field sites. For all countries, *P. falciparum* and *P. vivax* densities by us-qPCR were stratified according to a sample’s positivity by either of the qPCR assays or both assays. (Table 3 and Fig. 1c, d).

**Table 3:** *P. falciparum* and *P. vivax* densities stratified according to positivity by standard 18S rRNA qPCR (qPCR), by any qPCR, or only by ultra- sensitive qPCR (us-qPCR)

Country	*P. falciparum* Median Density^a^ Parasites/µL (IQR)			*P. vivax* Median Density^a^ *Pv*_mtCOX1 copies/µL (IQR)		
	qPCR( +)	qPCR( +) and us-qPCR( +)	qPCR(-) and us-qPCR( +)	qPCR( +)	qPCR( +) and us-qPCR( +)	qPCR(-) and us-qPCR( +)
Thailand	238.9 (55.8–1519)	40.8 (0.5–536.2)	0.2 (0.2–0.2)	104.8 (24.9–246.7)	50.4 (4.9–190.8)	3.1 (0.9–7.6)
Brazil	4.0^b^ (1.9–125.4)	0.4^b^ (0.2–3.0)	0.2 (0.1–0.3)	152.6 (50.3–738.7)	79.8 (18.3–464.4)	7.3 (3.7–22.0)
PNG	5.7 (1.0–70.0)	1.9 (0.2–22.5)	0.1 (0.0–0.4)	9.5 (3.1–145.9)	3.7 (0.9–16.4)	0.7 (0.3–1.2)

^a^Log_10_ transformed median parasite density quantified by us-qPCR

^b^Owing to exhaustion of DNA, parasite densities of 5 of the Brazilian samples were quantified by 18S rRNA qPCR

The median parasite density of *P. falciparum* infections detected by *Pf*_18S rRNA qPCR was highest in low-endemic Thailand, but significantly lower in low-endemic Brazil and moderate-endemic PNG (Table 3). This difference in median density may be attributed to symptomatic cases in the Thai sample set. When all symptomatic *P. falciparum* infections with densities > 1000 parasites/µL (3/15) were excluded from the analysis of Thai community samples, the median *P. falciparum* density decreased from 238.9 parasites/µL to 11.2 parasites/µL (IQR: 0.2–102.3 parasites/µL). These densities from asymptomatic Thai study participants are in the same range as densities observed in PNG and Brazil (Fig. 1c). In all three settings, parasite densities in infections detected only by us-qPCR were 10–20 times lower than densities in infections only positive by the less sensitive *Pf*_18S rRNA qPCR (p < 0.001) (Table 3).

Just as for *P. falciparum*, *P. vivax* parasite densities in infections only detected by us-qPCR were 8–10 times lower than densities in *P. vivax* infections detected by *Pv*_18S rRNA qPCR (p < 0.001) (Table 3, Fig. 1d). The median *P. vivax* parasite density in *P. vivax*-positives detected by *Pv*_18S rRNA qPCR was 11-fold lower in community samples from PNG than in those from Thailand, and 16-fold lower than in Brazil (p < 0.05) (Table 3). Similarly, the median *P. vivax* density in samples only positive by us-qPCR was significantly lower in PNG compared to Thailand and Brazil (p < 0.05) (Table 3).

### Age trends of *P. falciparum* and *P. vivax* infections

**Fig. 2:**
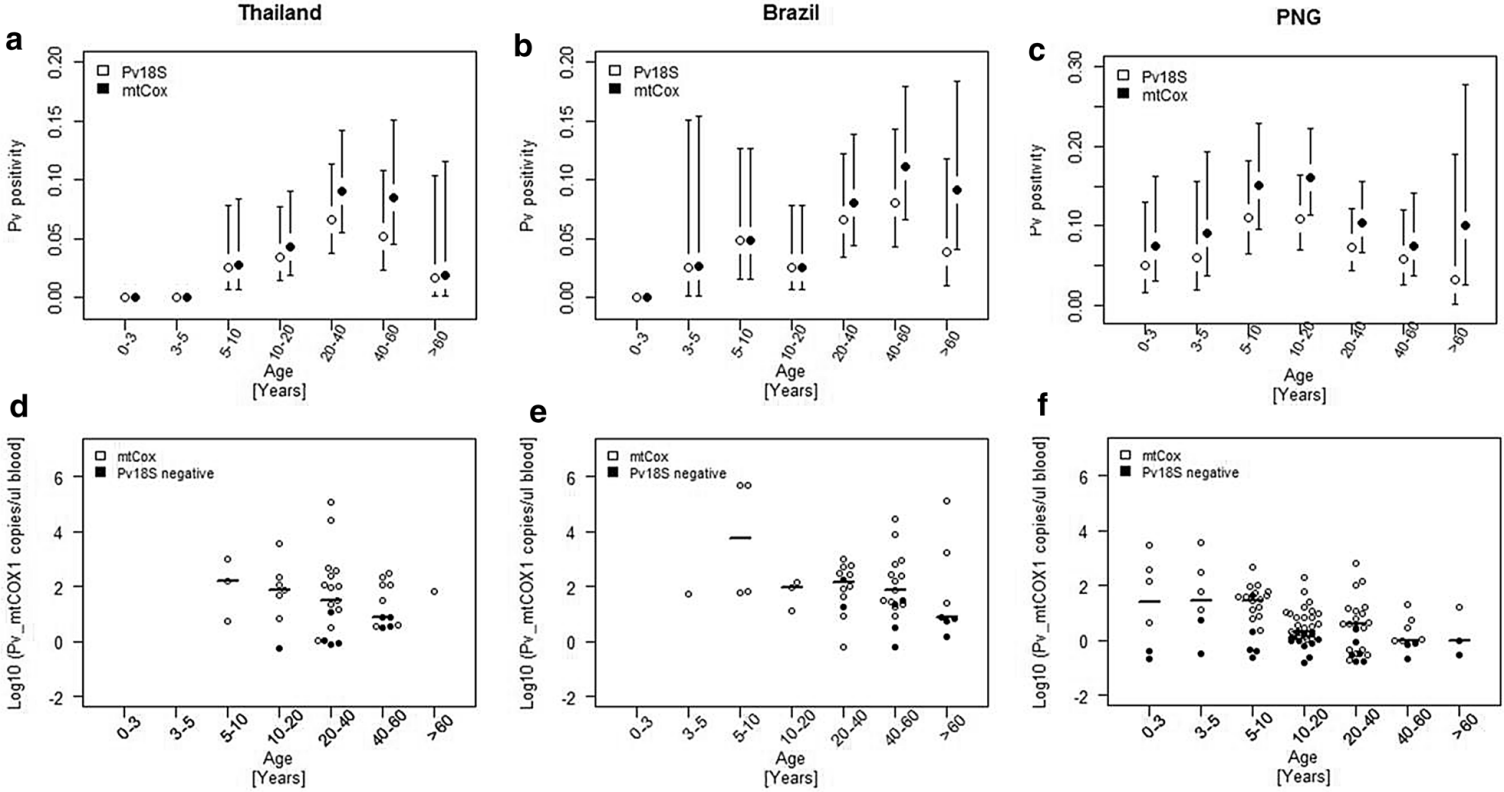
Age trends in *P. vivax* positivity and parasite density in Thailand, Brazil and PNG. *P. vivax* infections detected by *Pv_*18S rRNA qPCR and *Pv_*mtCOX1 us-qPCR (white circles) and *P. vivax* infections only detected by *Pv_*mtCOX1 us-qPCR (black circles). **a**–**c** *P. vivax* positivity by age group with 95% confidence intervals (black vertical lines). **d**–**f** Log_10_ transformed *P. vivax* parasite density by *Pv_*mtCOX1 us-qPCR per age group with median *Pv_mtCOX1* copy numbers (horizontal lines)

To identify population sub-groups that harbour particularly low-density infections and where benefits from us-qPCR would thus be greatest, parasite prevalence and density was analysed with respect to age. In all study sites, *P. vivax* positivity varied by age. This trend was consistent by both diagnostic assays. The age-stratified prevalence rates generated by the two molecular assays did not differ significantly at any study site (Chi^2^; Thailand: p-value = 0.96, Brazil: p = 0.79, PNG: p = 0.73). In Thailand and Brazil, *P. vivax* prevalence by both, standard and us-qPCR, peaked in adults aged 20–60 years (Fig. 2a, b**)**, whereas in PNG prevalence was highest by both assays in adolescents aged 10–20 years (Fig. 2c**)**. No *P. vivax* infection was found in the youngest age group in Brazil and Thailand (0–3 years), whereas *P. vivax* infections were detected by both assays in children from PNG aged 0–3 years (Fig. 2a–f). *Plasmodium vivax* infections were also absent in Thai children aged 3–5 years. At all three sites, median *P. vivax* densities measured by *Pv_*mtCox1 copies/µL blood were highest in the youngest age groups that included *P. vivax*-positive individuals, and tended to decrease by age (Fig. 2d–f). These trends were not statistically significant owing to the limited number of *P. vivax*-positive individuals in some age groups.

Age trends for *P. falciparum* prevalence and parasite density in Thailand and Brazil could not be analysed because of the limited number of *P. falciparum*-positive individuals. For PNG, the age distribution of *P. falciparum* infections is shown in Additional file 2: Fig. S4.

## Discussion

This study investigated the extent of low-density *P. falciparum* and *P. vivax* infections in moderate and low transmission settings in Thailand, Brazil and PNG, using ultra-sensitive detection assays. The LODs of the *P. falciparum* and *P. vivax* us-qPCR assays, which was 10-times lower than the standard assay *Pf*_18S rRNA qPCR [10, 11] were in good agreement between the three laboratories. The inter-laboratory comparability of results was closely controlled by the use of external reference standards. To evaluate the usefulness of us-qPCR assays for epidemiological studies, age trends in prevalence rates were analysed. No differences were observed in age-dependent epidemiological pattern in prevalence nor density in any transmission setting, thus confirming the suitability of us-qPCR for epidemiological research.

Substantial differences in gains were observed across transmission settings. For example, at the PNG study site, *P. falciparum* prevalence in the community increased from 8.6% to 12.2% by us-qPCR, an increase of 30% in *P. falciparum* positivity. In Brazil *P. falciparum* prevalence was much lower and a two-fold gain was observed in the proportion of positive samples by use of the ultra-sensitive varATS assay. However, the absolute numbers of *P. falciparum* infections in this comparative study were too low (i.e. a rise from 6/651 to 11/651 *P. falciparum*-positive individuals) so that the difference between study sites in the proportional gains was not statistically significant. The question whether proportional gains are higher at very low *P. falciparum* transmission intensity remains to be clarified by a larger study with a sufficient *P. falciparum* sample size for statistical analysis. Gains that differ by transmission intensity could occur if low-density *P. falciparum* infections undetectable by standard *Pf*_18S rRNA qPCR, would be relatively more abundant in low compared to high transmission. Earlier meta-analyses of global prevalence data of both, PCR- and microscopy-based diagnosis [1, 16] had revealed a negative relationship of the proportion of submicroscopic infections with transmission intensity.

The seemingly lower proportional gain in PNG compared to the Brazilian site (30% versus 55%) might be related to slightly higher overall *P. falciparum* parasite densities in community samples from PNG (1.9 parasites/µL) compared to Brazil (0.4 parasites/µL). The small difference in median *P. falciparum* densities between PNG and Brazil is in proximity to the LOD of the *Pf*_18S rRNA assay (1.57 parasites/µL blood) and therefore could have caused a higher proportion of infections in PNG samples to be detectable by standard *Pf*_18S rRNA qPCR. Whether density differences between sites could explain the smaller gains in PNG by using us-qPCR remains unclear, as the limited number of *P. falciparum* cases from low endemic Brazil precludes clarification of reasons for differing proportional gains.

Among all three sites, the *P. falciparum* median density was highest in Thailand with 238 parasites/µL compared to 4.0 parasites/µL in Brazil. This was due to very few high density infections with > 1000 parasites/µL in this community. In addition, 3 of these *P. falciparum*-infected cases showed evidence of fever within the preceding 2 days. These findings from Thailand could mirror declining transmission and indicate that less frequent exposure to *P. falciparum* may result in waning immunity against this parasite. This would suggest that *P. falciparum*-infected individuals at the Thai study site more often develop high parasite densities and become symptomatic and thus will receive treatment. It, therefore, can be speculated that the presence of malaria episodes has caused a lower proportional gain of infections detected by us-qPCR in Thailand compared to low-endemic Brazil.

*Pv*_mtCOX us-qPCR resulted in an analogical increase in the proportion of additionally detected *P. vivax*-infections across all study sites, ranging from 31% in PNG to 24% in Brazil and 23% in Thailand. Compared to the more pronounced differences in *P. falciparum* proportional gains, the *Pv*_mtCOX-based increase was similar across the 3 sites. When *P. vivax* median densities were consulted to explain the small difference in proportional gains between study sites, 11–16-fold higher *P. vivax* densities were observed in Brazil and Thailand compared to PNG. This might be responsible for the lower gain by more sensitive diagnostics in these two low endemic settings.

Benefits from us-qPCR consist in the improved detection of low-density *P. falciparum* and *P. vivax* infections. The question arises, whether these low-density infections carry gametocytes in sufficient numbers to contribute to onward transmission to mosquitoes. This question has been addressed by previous studies at all three sites, either by membrane feeding assays on symptomatic blood samples [20, 21] or by using gametocyte density as surrogate for transmission potential, such as in a study recently performed in the same community in PNG, where gametocytes were quantified after enrichment from large volumes of blood [12]. In this recent study, more than half of the *P. falciparum-* and *P. vivax*-positive samples that were only detected by us-qPCR, carried gametocytes, (10 gametocyte carriers in 15 *P. falciparum*-infections and 11 gametocyte carriers in 19 *P. vivax*-infections) [12]. Gametocyte densities were tenfold lower in infections detected by standard 18S rRNA qPCR compared to infections only detectable by us-qPCR and were below 1 male and 1 female gametocyte/µL [22]. Although the gametocyte sex-ratio suggested a very low likelihood of transmission [22], it cannot be excluded that parasite densities in untreated, chronic malaria infections in the community may fluctuate and transiently reach levels that could potentially be infective to mosquitoes [23].

A limitation in the quantification of *P. vivax* infections derives from differences in the numbers of mitochondria between different *P. vivax* blood stages, i.e. between a ring stage *versus* a multi-nucleated schizont stage, both present in peripheral blood. The number of target gene copies per parasite varies accordingly [10] [17]. *Plasmodium vivax* parasite densities calculated from qPCR copy numbers thus represent only an estimate. Yet, this equally applies to the variable number of nuclear genomes and thus to 18S rDNA copies circulating in *P. vivax* parasites of any stage. This explains our observation of a good correlation between both markers for quantification of *P. vivax*. For other human *Plasmodium* species occurring at the 3 study sites, us-qPCR assays have not yet been developed, thus the analyses performed in this study were limited to *P. falciparum* and *P. vivax*.

Investigations of individuals at greatest risk of infection based on parasite detection by *Pf*_ and *Pv*_18S rRNA qPCR assays have been described previously for all three study sites [19, 24] [W. Monteiro, personal communication]. These earlier analyses showed that main predictors of *P. falciparum* and *P. vivax* infections and density differed substantially between sites. To complement these former epidemiological analyses, age trends of *P. falciparum* and *P. vivax* infection in relation to parasite densities based on us-qPCR data were investigated. Parasite carriers with very low-density infections in PNG were primarily found in adolescents aged 10–20 years, whereas in Thailand and Brazil most additionally gained infections were found in older individuals (> 40 years of age). Therefore, the additional *P. vivax* infections detected by us-qPCR coincided with the age group that had presented the highest risk of infection in earlier analyses from Thailand, Brazil and PNG [19, 24] (W.M. Monteiro, pers. commun.).

Depending on the public health question to be addressed by molecular diagnosis, more precise prevalence data as achieved by us-qPCR may be of considerable relevance, particularly in low transmission settings, where each individual malaria case is investigated by elimination programmes [25]. Furthermore, results are likely to be more consistent across surveys, as stochastic amplification of low-density infections is reduced [2, 11]. Because this study demonstrated a substantial gain of additionally detected low-density infections, particularly in low transmission settings, us-qPCR could be crucial for surveillance in elimination settings. Molecular diagnosis is increasingly used to determine presence of infections to guide control efforts [26]. The use of us-qPCR, and thus greater confidence in diagnostic metrics, could reduce the number of samples to be screened before declaring a region malaria-free.

## Conclusion

The use of us-qPCR for parasite detection in the community provided a more accurate picture of low-density infections at different levels of transmission intensity. The benefits of using ultra-sensitive assays were clearly demonstrated for all study sites. When analysing the relationship of low versus high transmission intensity and the gain in *P. vivax* positivity by us-qPCR, only minor differences were observed between study sites in the *P. vivax* proportional gains in positivity that ranged from 23% to 31%. The *P. falciparum* proportional gain was almost twice as high in Brazil (low *P. falciparum* transmission) than in PNG (high *P. falciparum* transmission). However, this difference was not statistically significant, as the absolute number of *P. falciparum* positive samples in the Brazilian community survey was as low as 1.7% (11/651) by *P. falciparum* us-qPCR. Further comparative studies are required to clarify a potentially higher gain in *P. falciparum* positivity in low transmission versus high transmission.

In areas of low *P. falciparum* and *P. vivax* transmission, the absolute number of infections additionally gained by ultra-sensitive methods seem few. However, for certain research questions arising in pre-elimination settings, such increased sensitivity might be beneficial for informing control measures. For instance, *Pv*_us-qPCR can play an important role in both epidemiological studies and surveillance, because those additionally detected *Pv*-positive individuals may experience relapses and in future contribute to onwards transmission.

## Supplementary information

**Additional file 1.** Supplementary Table.

**Additional file 2.** Supplementary Figures.

## Data Availability

All data presented in this article or accompanying Additional files can be made available upon request.

## Abbreviations

DNA: Deoxyribonucleic acid
LM: Light microscopy
LOD: Limit of detection
mtCOX1: Mitochondrial cytochrome C oxidase 1
PCR: Polymerase chain reaction
PNG: Papua New Guinea
qPCR: Quantitative polymerase chain reaction
RDT: Rapid diagnostic test
us: Ultra-sensitive
*var*ATS: *Var* gene acidic terminal sequence
