# Supplementary material for: Utility of ultra-sensitive qPCR to detect Plasmodium falciparum and Plasmodium vivax infections under different transmission intensities
Source: Malar J. 2020 Sep 3;19:319. doi: 10.1186/s12936-020-03374-7 (PMC7469345; doi:10.1186/s12936-020-03374-7)
Supplement: Supplementary file 1 — Additional file 1. Supplementary Table. [file 12936_2020_3374_MOESM2_ESM.pdf]

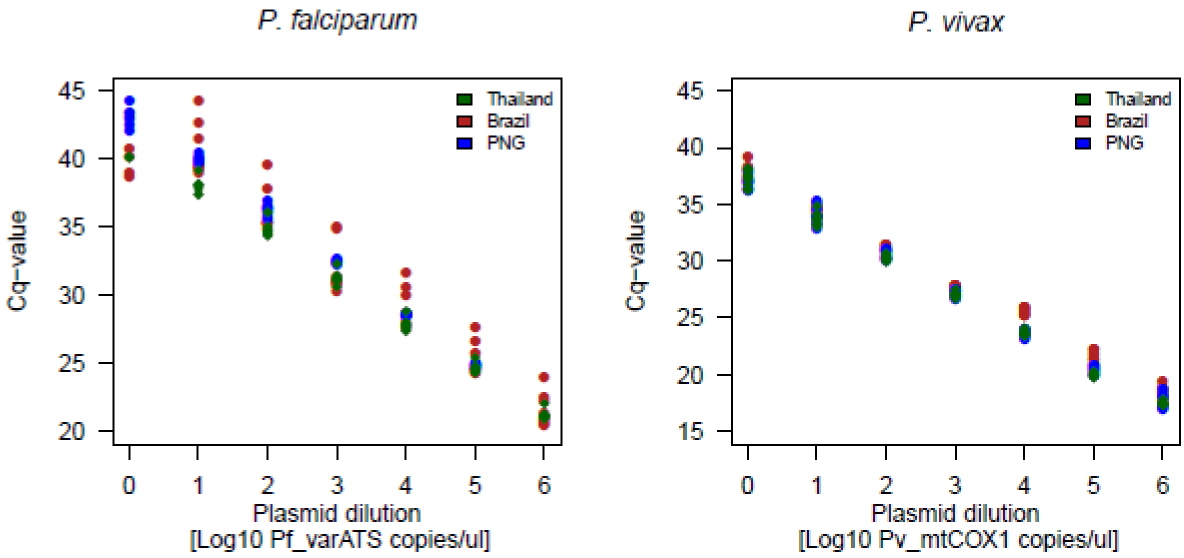

Fig. S1: Comparison of *Pf*_varATS and *Pv*_mtCOX1 plasmid standards across the 3 study sites.

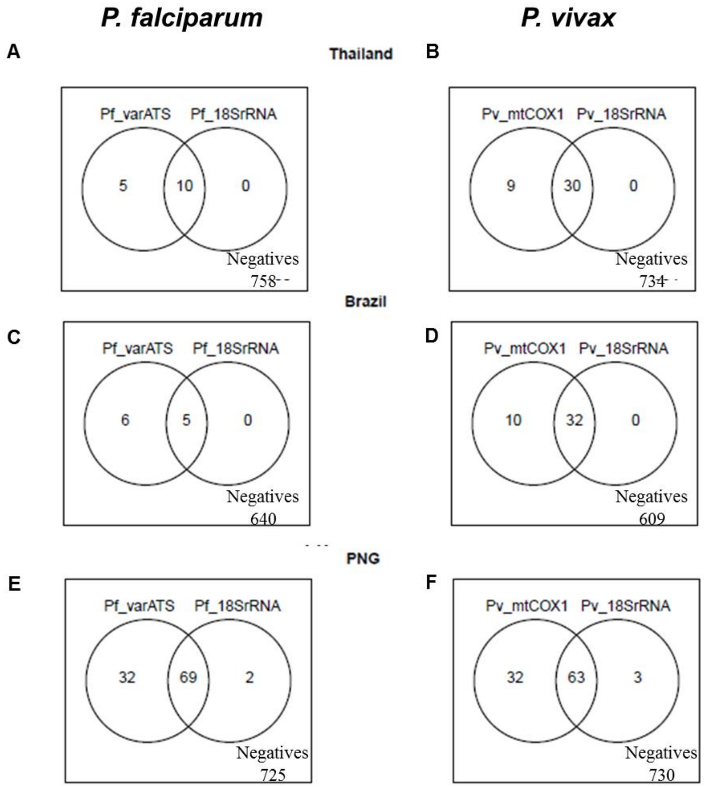

Fig. S2: Venn diagrams of *P. falciparum* and *P. vivax* positivity by ultra-sensitive and standard 18SrRNA qPCR.

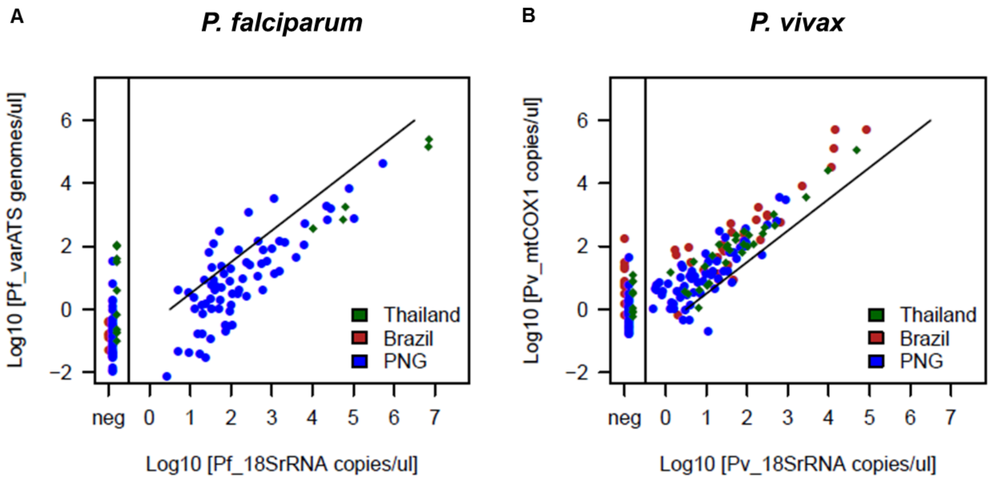

Fig. S3: Correlation of *P. falciparum* (A) and *P. vivax* (B) densities between ultra-sensitive and standard 18srRNA qPCR. Spearman's correlation coefficient was assessed on $\log_{10}$-transformed parasite densities for *P. falciparum*: rho(PNG) = 0.74, *P. vivax*: rho (PNG) =0.80 , rho(Brazil)=0.85, rho(Thailand)=0.94. Because of only few *Pf*-positives in the data sets from Thailand and Brazil, the correlation between assays was not assessed for these study sites. Black line denotes perfect correlation (r=1).

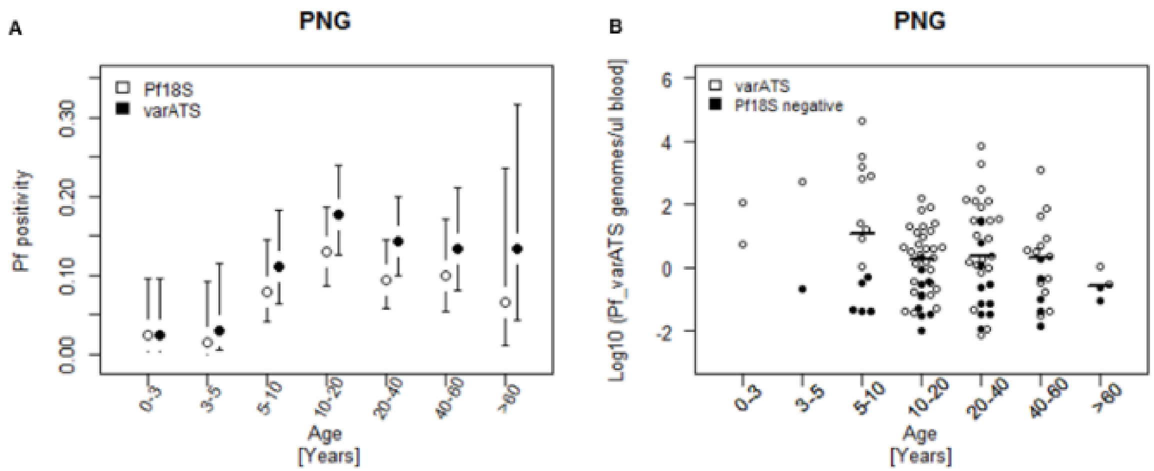

Fig. S4: Age trends in *P. falciparum* positivity rates (A) and parasite densities (B) in PNG. *Pf* positivity by *Pf*_18SrRNA qPCR (white circles) and *Pf*_varATS us-qPCR (black circles) stratified by age group. Black vertical lines in panel A denote 95% confidence interval. Panel B represents *Pf_varATS* copy numbers per age group. Median copy numbers are denoted (horizontal lines).
