# Supplementary material for: Utility of ultra-sensitive qPCR to detect Plasmodium falciparum and Plasmodium vivax infections under different transmission intensities
Source: Malar J. 2020 Sep 3;19:319. doi: 10.1186/s12936-020-03374-7 (PMC7469345; doi:10.1186/s12936-020-03374-7)
Supplement: Supplementary file 2 — Additional file 2. Supplementary Figures. [file 12936_2020_3374_MOESM1_ESM.pdf]

Table S1: Performance details of ultra-sensitive *P. falciparum* and *P. vivax* qPCR assays and efficiencies of plasmid standard trendlines generated at 3 different study sites.

Assay	Country	Slope	Intercept	Efficiency	R2
*Pf*_varATS	Thailand	-3.3	41.1	101.0%	0.99
*Pf*_varATS	Brazil	-3.5	42.8	93.1%	0.95
*Pf*_varATS	PNG	-3.7	43.5	86.3%	1
*Pv*_mtCOX1	Thailand	-3.3	37.1	101.0%	1
*Pv*_mtCOX1	Brazil	-3.2	37.7	105.4%	0.99
*Pv*_mtCOX1	PNG	-3.3	37.1	101.0	0.99
